# Supplementary figures and images for: Antigen flexibility supports the avidity of hemagglutinin-specific antibodies at low antigen densities
Source: PLoS Pathog. 2026 Feb 5;22(2):e1013862. doi: 10.1371/journal.ppat.1013862 (PMC13020973; doi:10.1371/journal.ppat.1013862)

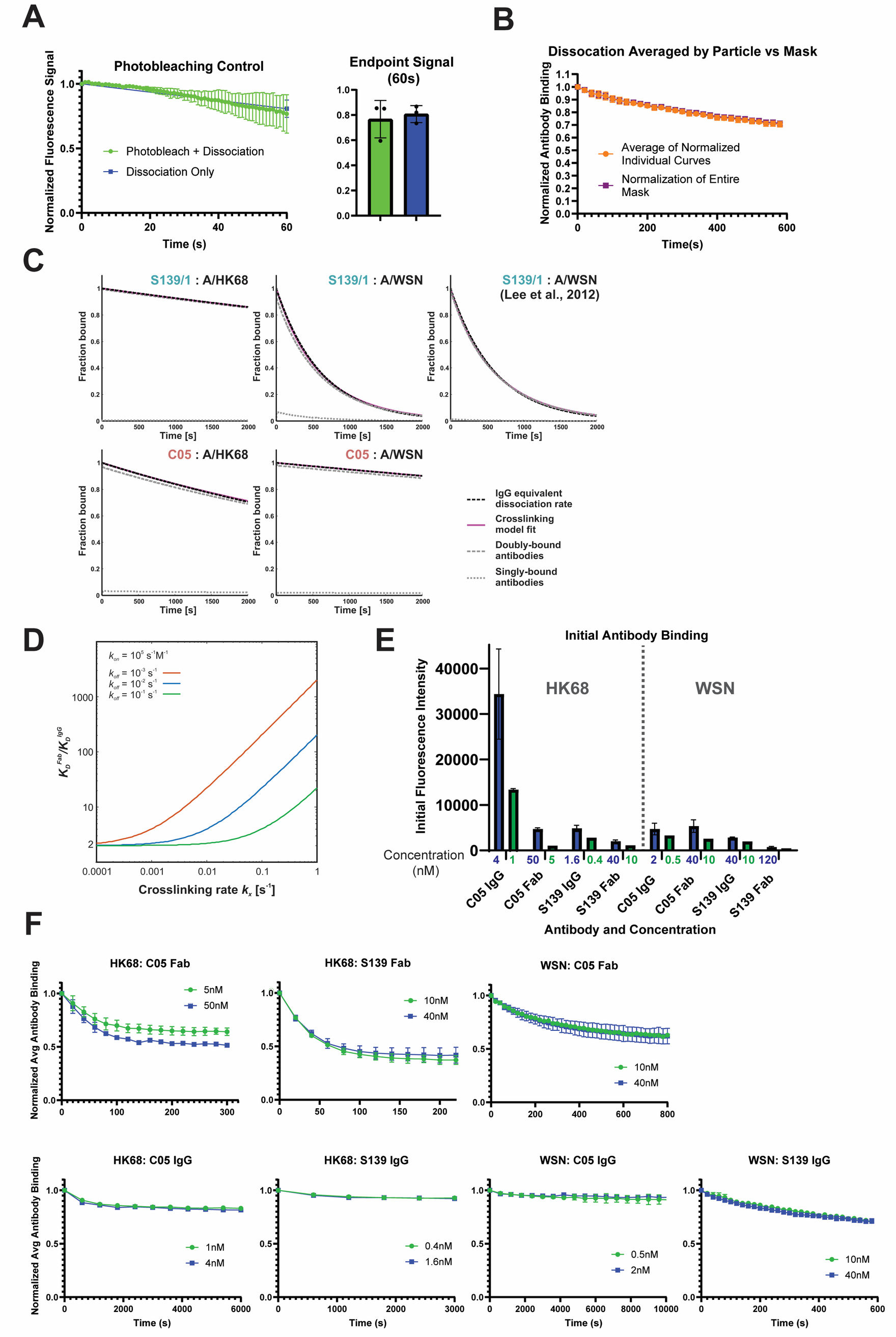

Supplement: S1 Fig — (A) To control for photobleaching, the loss of fluorescence signal was imaged under two conditions: 1) dissociation only; an image was collected at 0s and one at 60s and 2) dissociation and photobleaching; an image was collected at a rate of 1 frame per second for 60 seconds. The difference between the endpoint intensities from both conditions is not statistically significant. (B) To account for any differences in weighting the average, the normalized dissociation curves were averaged in two ways: 1) by taking the average with each virion equally weighted, and 2) with the entire field of view masked and normalized together. (C)To fit the crosslinking rate (kx), the experimentally obtained koff values from each Fab and IgG pair were mapped to the simulation results. For a given Fab koff value, we iterate through a series of kx values and determine the best fit to the dissociation curve for the corresponding IgG. (D) The fold difference in effective koff is shown as a function of kx for a few given Fab koff values. (E) Intensities of bound antibodies prior to measuring dissociation. Data is from the experiments shown normalized in Fig 1C. The concentration for each antibody is shown in blue or green for the higher and lower concentrations, respectively (F) Normalized dissociation curves for the conditions plotted in D. (TIF) [file ppat.1013862.s001.tif]

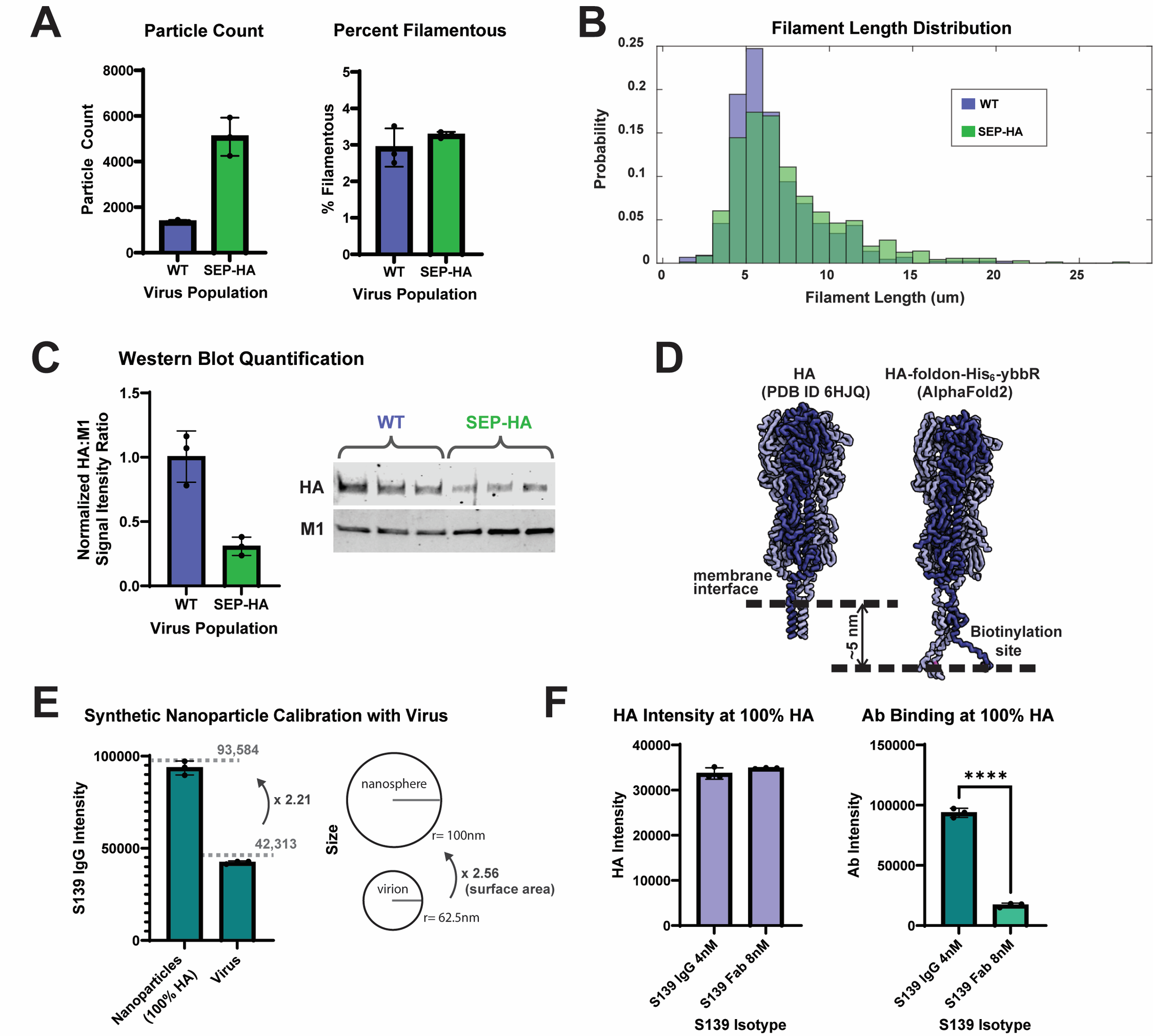

Supplement: S2 Fig — Comparisons of wildtype and SEP-HA virus populations based upon (A) confocal microscopy analysis of the total particle count and percentage of the virion population comprised of filamentous particles; and (B) filament length distributions, and (C) a quantification of Western blot intensity values of relative HA signal normalized by M1 in each supernatant population; HA and M1 are quantified on the same gel by different antibodies. (D) Comparison of the structures of native HA (PDB ID 6HJQ) and the recombinant HA with trimerization domain and linker used in synthetic nanoparticle experiments (AlphaFold2). (E) Calibration of HA density on streptavidin nanoparticles relative to native virions. Plot to the left quantifies the fluorescent intensity of labeled S139/1 IgG bound to nanoparticles (incubated with biotinylated HA) and viruses. Schematic to the right shows relative sizes of nanoparticles and virions used to calculate relative HA densities. The corresponding scaling between the size of an average spherical virion to a nanoparticle is shown. (F) Quantification of HA intensities (left plot) and antibody intensities (right plot) for beads with saturating densities (‘100%’) of HA, incubated with S139/1 IgG or S139/1 Fab. (TIF) [file ppat.1013862.s002.tif]

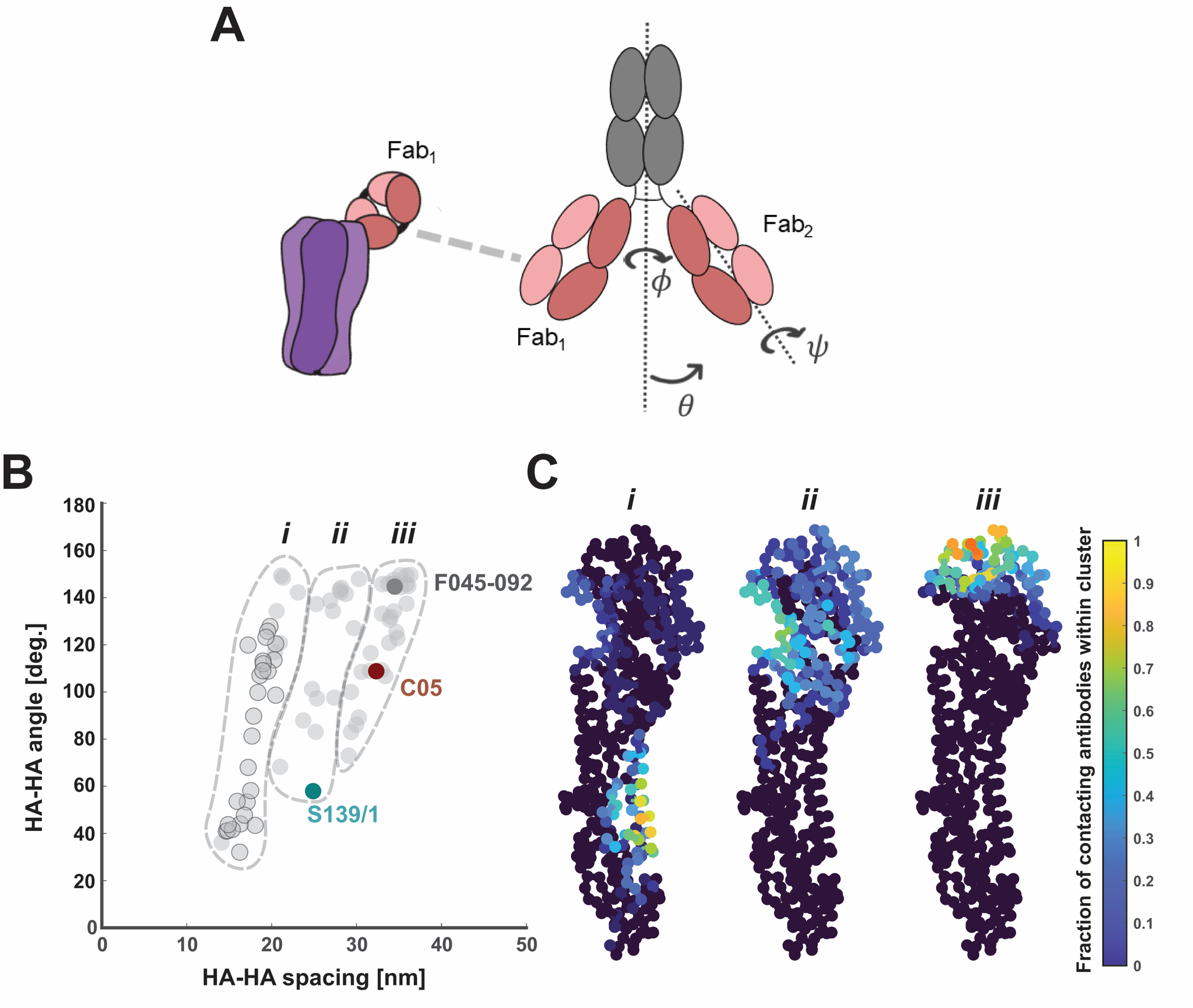

Supplement: S3 Fig — (A) Schematic representing the geometric binding model. For a given HA-Fab interaction, the first Fab arm (Fab1) is aligned to its binding site on HA. The position of the second arm is simulated by sampling conformations about the degrees of freedom indicated. (B) Predictions from the structure-based model from Fig 4A, extended to structures of HA-antibody complexes from the PDB. Each point represents the most frequent inter-HA spacing and angle samples by a particular antibody. S139/1, C05, and F045-092, another high-avidity antibody, are highlighted. Stem-binding antibodies are indicated by a black outline. (C) Structure of an HA monomer with each residue colored according to the frequency of contacts by antibodies in the corresponding groups from the plot in A. Contacts are defined as HA residues within 0.8 nm of any residue from the antibody heavy or light chain. (TIF) [file ppat.1013862.s003.tif]
