# Supplementary material for: Antigen flexibility supports the avidity of hemagglutinin-specific antibodies at low antigen densities
Source: PLoS Pathog. 2026 Feb 5;22(2):e1013862. doi: 10.1371/journal.ppat.1013862 (PMC13020973; doi:10.1371/journal.ppat.1013862)
Supplement: S1 Text — (DOCX) [file ppat.1013862.s006.docx]

**Sequences for Proteins Used in Antibody Binding Experiments**

S139/1 HC:

MGWSCIILFLVATATGVHSEVQLQQSGTELKKPGASVKISCKATGYTFSSYWIEWIKQRPGHGLEWIGEILPEIGMTNYNENFKGKATFTANTSSNTVYMQLSSLTSEDSAVYYCARPYDYSWFAYWGQGTLVTVSSASTKGPSVFPLAPSSKSTSGGTAALGCLVKDYFPEPVTVSWNSGALTSGVHTFPAVLQSSGLYSLSSVVTVPSSSLGTQTYICNVNHKPSNTKVDKRVEPKSCDKTHTCPPCPAPELLGGPSVFLFPPKPKDTLMISRTPEVTCVVVDVSHEDPEVKFNWYVDGVEVHNAKTKPREEQYNSTYRVVSVLTVLHQDWLNGKEYKCKVSNKALPAPIEKTISKAKGQPREPQVYTLPPSRDELTKNQVSLTCLVKGFYPSDIAVEWESNGQPENNYKTTPPVLDSDGSFFLYSKLTVDKSRWQQGNVFSCSVMHEALHNHYTQKSLSLSPGK*

S139/1 HC Fab:

MGWSCIILFLVATATGVHSEVQLQQSGTELKKPGASVKISCKATGYTFSSYWIEWIKQRPGHGLEWIGEILPEIGMTNYNENFKGKATFTANTSSNTVYMQLSSLTSEDSAVYYCARPYDYSWFAYWGQGTLVTVSSASTKGPSVFPLAPSSKSTSGGTAALGCLVKDYFPEPVTVSWNSGALTSGVHTFPAVLQSSGLYSLSSVVTVPSSSLGTQTYICNVNHKPSNTKVDKRVEPKSCDKGGSHHHHHHGGSDSLEFIASKLA*

S139/1 LC:

MGWSCIILFLVATATGVHSDIVMTQSQKFMSTSVGDRVSVTCKASQNVDTNVAWYQEKPGQSPKTLIYSASNRYSGVPDRFTGSASGTDFTLTITNVQSEDLAEYFCQQYNSYPYTFGGGTKLEIKRADAAPSVFIFPPSDEQLKSGTASVVCLLNNFYPREAKVQWKVDNALQSGNSQESVTEQDSKDSTYSLSSTLTLSKADYEKHKVYACEVTHQGLSSPVTKSFNRGEC*

C05 HC:

MGWSCIILFLVATATGVHSEVQLQESGGGLVQPGESLRLSCVGSGSSFGESTLSYYAVSWVRQAPGKGLEWLSIINAGGGDIDYADSVEGRFTISRDNSKETLYLQMTNLRVEDTGVYYCAKHMSMQQVVSAGWERADLVGDAFDVWGQGTMVTVSSASTKGPSVFPLAPSSKSTSGGTAALGCLVKDYFPEPVTVSWNSGALTSGVHTFPAVLQSSGLYSLSSVVTVPSSSLGTQTYICNVNHKPSNTKVDKRVEPKSCDKTHTCPPCPAPELLGGPSVFLFPPKPKDTLMISRTPEVTCVVVDVSHEDPEVKFNWYVDGVEVHNAKTKPREEQYNSTYRVVSVLTVLHQDWLNGKEYKCKVSNKALPAPIEKTISKAKGQPREPQVYTLPPSRDELTKNQVSLTCLVKGFYPSDIAVEWESNGQPENNYKTTPPVLDSDGSFFLYSKLTVDKSRWQQGNVFSCSVMHEALHNHYTQKSLSLSPGK*

C05 HC Fab:

MGWSCIILFLVATATGVHSEVQLQESGGGLVQPGESLRLSCVGSGSSFGESTLSYYAVSWVRQAPGKGLEWLSIINAGGGDIDYADSVEGRFTISRDNSKETLYLQMTNLRVEDTGVYYCAKHMSMQQVVSAGWERADLVGDAFDVWGQGTMVTVSSASTKGPSVFPLAPSSKSTSGGTAALGCLVKDYFPEPVTVSWNSGALTSGVHTFPAVLQSSGLYSLSSVVTVPSSSLGTQTYICNVNHKPSNTKVDKRVEPKSCDKGGSHHHHHHGGSDSLEFIASKLA*

C05 LC:

MGWSCIILFLVATATGVHSDIQLTQSPSSLSASVGDRVTLTCQASQDIRKFLNWYQQKPGKGPKLLIYDASNLQRGVPSRFSGGGSGTDFTLIISSLQPEDVGTYYCQQYDGLPFTFGGGTKVVIKRTVAAPSVFIFPPSDEQLKSGTASVVCLLNNFYPREAKVQWKVDNALQSGNSQESVTEQDSKDSTYSLSSTLTLSKADYEKHKVYACEVTHQGLSSPVTKSFNRGEC*

FISW84 Fab HC:

MGWSCIILFLVATATGVHSEVQLLESGGGLVQPGGSLRLSCAASGFTFSSYGMAWVRQAPGKGLEWVSFISATGLSTYFADSVKGRFTISRDTTKNTLYLQMNSLRADDTAVYFCARMRRTMIAFGGNDFWGQGTLVTVSSASTKGPSVFPLAPSSKSTSGGTAALGCLVKDYFPEPVTVSWNSGALTSGVHTFPAVLQSSGLYSLSSVVTVPSSSLGTQTYICNVNHKPSNTKVDKRVEPKSCDKGGSHHHHHHGGSDSLEFIASKLA*

FISW84 LC:

MGWSCIILFLVATATGVHSEVVMTQSPATLSVSPGEGATLSCRASQSVNTNVAWYQQKPGQAPRLLIYGASTRATGIPARFSGSGSGTEFTLTISTLQSEDFAVYYCQQYSNWPPITFGQGTRLEIKRTVAAPSVFIFPPSDEQLKSGTASVVCLLNNFYPREAKVQWKVDNALQSGNSQESVTEQDSKDSTYSLSSTLTLSKADYEKHKVYACEVTHQGLSSPVTKSFNRGEC*

A/Hong Kong/1968 HA ectodomain:

MKTIIALSYIFCLALGQDLPGNDNSTATLCLGHHAVPNGTLVKTITDDQIEVTNATELVQSSSTGKICNNPHRILDGIDCSLIDALLGDPHCDVFRNETWDLFVERSKAFSNCYPYDVPDYASLRSLVASSGTLEFITEGFTWTGVTQNGGSNACKRGPGSGFFSRLNWLTKSGSTYPVLNVTMPNNDNFDKLYIWGVHHPSTNQEQTSLYVQASGRVTVSTRRSQQTIIPNIGSRPWVRGLSSRISIYWTIVKPGDVLVINSNGNPIAPRGYFKMRTGKSSIMRSDAPIDTCISECITPNGSIPNDKPFQNVNKITYGACPKYVKQNTLKLATGMRNVPEKQTRGLFGAIAGFIENGWEGMIDGWYGFRHQNSEGTGQAADLKSTQAAIDQINGKLNRVIEKTNEKFHQIEKEFSEVEGRIQDLEKYVEDTKIDLWSYNAELLVALENQHTIDLTDSEMNKLFEKTRRQLRENAEDMGNGCFKIYHKCDNACIESIRNGTYDHDVYRDEALNNRFQIKGVELKSGYKDGYIPEAPRDGQAYVRKDGEWVLLSTFLGSGSHHHHHHGGDSLEFIASKLA*

SEP-HA (A/WSN/1933):

MVEMLPTVAVLVLAVSVVAKDNTTLQEFATMVKGEELFTGVVPILVELDGDVNGHKFSVSGEGEGDATYGKLTLKFICTTGKLPVPWPTLVTTLTYGVQCFSRYPDHMKRHDFFKSAMPEGYVQERTIFFKDDGNYKTRAEVKFEGDTLVNRIELKGIDFKEDGNILGHKLEYNYNDHQVYIMADKQKNGIKANFKIRHNIEDGGVQLADHYQQNTPIGDGPVLLPDNHYLFTTSTLSKDPNEKRDHMVLLEFVTAAGITHGMDELYKGGENLYFQGGGGSKQIEDKIEEILSKIYHIENEIARIKKLIGGSGVYQILAIYSTVASSLVLLVSLGAISFWMCSNGSLQCRICI*
