# Supplementary material for: Antigen flexibility supports the avidity of hemagglutinin-specific antibodies at low antigen densities
Source: PLoS Pathog. 2026 Feb 5;22(2):e1013862. doi: 10.1371/journal.ppat.1013862 (PMC13020973; doi:10.1371/journal.ppat.1013862)
Supplement: Attachment — Submitted filename: Response to Reviewers.pdf [file ppat.1013862.s008.pdf]

We thank the Reviewers for their thoughtful evaluation of our work. Based on their suggestions, we have revised Figure S3 to highlight antibodies that bind to the HA stem, and we have included the simulated data in tabular form within the supplemental file S2. More details are given in our point-by-point response below.

Reviewer #1: Regarding Figure S3, I noticed that cluster 'i' appears to contain nearly all of the HA stem antibodies, plus a few from the head region. Would it be possible to highlight which data points within this cluster correspond specifically to the stem antibodies?

The reviewer's observation is correct; the majority of antibodies in cluster 'i' bind to the HA stem. Moreover, we find that all of the stem binding antibodies reside within this cluster. To better highlight this observation, data points corresponding to stem binding antibodies are now displayed with a black outline in the revised Figure S3.

Furthermore, while this modeling is in the supplemental material, the dataset itself is quite valuable. It would be a great benefit to the field if the authors could make this data available to readers, perhaps in a new supplementary table listing each antibody with its most frequent inter-HA spacing and angle and/or in the authors' GitHub link.

To make this data more accessible to anyone who is interested, we have added a new supplemental table that lists all of the antibodies analyzed by PDB ID, along with the typical inter-HA spacing and angle. These entries are also annotated to indicate if the antibody binds to the HA stem.

Of note, it also looks like the authors' Github link is still not working, which they may want to fix for the final version of the manuscript.

Thank you for calling this to our attention. We have now made the Github link from the article public.

Reviewer #2: None noted, the data are clearly presented.

Reviewer #3: All my previous comments and those of other reviewers were adequately addressed.
