# Supplementary material for: Antigen flexibility supports the avidity of hemagglutinin-specific antibodies at low antigen densities
Source: PLoS Pathog. 2026 Feb 5;22(2):e1013862. doi: 10.1371/journal.ppat.1013862 (PMC13020973; doi:10.1371/journal.ppat.1013862)
Supplement: Attachment — Submitted filename: 152957_1_rebuttal_3429158_sy7r6s.docx [file ppat.1013862.s007.docx]

**Manuscript number:** RC-2024-02804

**Corresponding author(s):** Vahey, Michael

## 1. General Statements

We thank the reviewers for their thoughtful and constructive feedback, which we believe has significantly strengthened this work. While the reviewers were enthusiastic about the manuscript, they raised a number of points which we have clarified and expanded upon through new experimental data, further analysis, and revisions to the text and figures. To this end, we have:

- Added a more robust control for photobleaching during dissociation experiments
- Incorporated a western blot to validate HA content across virus populations, to complement microscopy data
- Included additional structural modeling information and updated methods for the geometric model of crosslinking
- Revised several figures and captions for clarity, and corrected references and links

## 2. Point-by-point description of the revisions

**Reviewer comments are shown in black, while our responses are shown in blue text.**

**Reviewer #1 (Evidence, reproducibility and clarity (Required)):**

Comments for the authors of Review Commons Manuscript RC-2024-02804:

The author of the Review Commons manuscript "Antigen flexibility supports the avidity of hemagglutinin-specific antibodies at low antigen densities", present their recent work evaluating hemagglutinin interactions with cellular receptors and antibodies. This manuscript focuses specifically on the avidity of the hemagglutinin using a fluorescence-based assay to measure dissociation kinetics and steady-state binding of antibodies to virions. Thie findings confirm that bivalent interactions can offset weak monovalent affinity and that HA ectodomain flexibility is an additional determinant of antibody avidity. These findings are key for our understanding of neutralizing antibodies. Below are some comments that I would like the authors to address as they revise the manuscript.

Comments:

1. Can the authors provide justification for the two influenza viruses that they used.

We selected the lab-adapted IAV strains A/WSN/1933 (H1N1) and A/Hong Kong/1968 (H3N2) for this work because they are well-studied, including in the context of the antibodies used here, S139/1 and C05. While both antibodies bind to more contemporary H3N2 strains, they no not bind to HA from pandemic H1N1. Another feature of these strains is that their HAs have high enough affinity to both antibodies to enable strong signal in our imaging assays. This context for our strain selection has been added in lines 85-88.

2. The use of filamentous particles is a strength, but authors should detail the role of filamentous vs. spherical in nature and lab settings. This will help researchers that plan to repeat these assays.

We have revised the text (lines 336-339) to include more context on the biology of filamentous and spherical influenza viruses. In our experiments, HK68 naturally produces filaments in cell culture whereas WSN33 does not. To produce filaments artificially, we replace the M1 sequence from WSN33 with that of M1 from A/Udorn/1972, an H3N2 strain that is closely related to HK68.

3. Did the authors add the Udorn M1 to the HK68 as well?

Since HK68 naturally forms filaments, we did not introduce Udorn M1 into this strain. We note that the amino acid sequences of Udorn M1 and HK68 M1 differ only at position 167 (Alanine in Udorn, Threonine in HK68), and that this residue has previously been found to not correlate with virus morphology (10.1016/j.virol.2003.12.009).

Reviewer #1 (Significance (Required)):

This manuscript focuses specifically on the avidity of the hemagglutinin using a fluorescence-based assay to measure dissociation kinetics and steady-state binding of antibodies to virions. Thie findings confirm that bivalent interactions can offset weak monovalent affinity and that HA ectodomain flexibility is an additional determinant of antibody avidity. These findings are key for our understanding of neutralizing antibodies.

**Reviewer #2 (Evidence, reproducibility and clarity (Required)):**

Summary

In this study, Benegal et al. investigate the binding kinetics of HA-head-specific antibodies (S139/1 and C05) to intact influenza virus particles using a fluorescence microscopy-based technique to measure the dissociation rate (koff) of the antibodies. By applying their proposed equilibrium model for bivalent antibody binding to HA, the authors calculated the crosslinking rate (kx), which represents the rate at which a single-bound antibody crosslinks to an additional HA molecule. Their experiments revealed that antigen crosslinking significantly slows koff, reducing it by up to two orders of magnitude.

The authors further utilized streptavidin-coated beads conjugated with biotinylated HA or biotinylated BSA at varying concentrations to control HA surface density. Their results demonstrated that the two tested HA-head-specific antibodies retained the ability to crosslink HAs even at ~10-fold lower HA surface densities. In a complementary experiment, they employed an HA-anchor-specific antibody to restrict HA flexibility, which led to reduced binding of S139/1 and C05 IgGs but not their Fab fragments. This finding suggests that HA flexibility, rather than density, is the primary determinant of antibody crosslinking and avidity.

Overall, the authors present an innovative approach to elucidating the dissociation and crosslinking kinetics of antibodies targeting intact virions or nanoparticles. The study is well-designed, with alternative interpretations of the results carefully considered and addressed throughout. I have only a few minor comments and suggestions for clarification.

Minor comments:

1. In Figure 1, does the grey color of each IgG in panel C indicate the Fc domain? If so, please add the description of the colors to the figure legend. In fact, it may be better to explain all the colors used here (for HA1, HA2, Fab heavy chain, light chain, etc.).

We have included this information in panel C and the caption for Figure 1.

2. Under the section," Bivalent binding of S139/1 and C05 persists after ~10-fold reductions in HA surface densities", the beginning of the second paragraph writes, "For both S139/1 and C05 Fab, binding increases linearly with HA density, as expected for a monovalent interaction dictated by absolute HA availability rather than density (Fig. 3D). Interestingly, the same relationship is observed for S139/1 IgG."

Visually, I think the same relationship also seems to hold for C05 IgG. Would it be better to perform some linear regression and report the R2 value for the fitting so that this assessment can be quantitative?

We agree with the reviewer’s point. In Figure 3 of the revised manuscript, we include the results from a linear regression analysis to make this assessment more quantitative.

3. At the end of the same page, in the same paragraph, the authors mentioned, "In contrast to the IgG, Fab binding measured at twice the molar concentration of the IgG is nearly undetectable under these conditions, confirming the IgG binding is not occurring through monovalent interactions (Fig. S2E)." What are the conditions you are referring to? In Fig. S2E, there is only the Ab intensity for the Ab binding at 100% HA (and not the other percentages). For the Ab intensity of S139/1 Fab, what is the concentration of the Fab used in Figure 3D? Why could the intensity in this experiment for S139/1 Fab reach ~100,000, whereas that of the 8 nM in Fig. S2E can only reach ~20,000?

To clarify this point, we have updated Figure 3 to include the antibody concentration used for each experiment. The experiments in Fig 3 are conducted approximately around the respective K_D_ of each IgG or Fab to ensure both consistency and strong signal-to-noise. For S139/1, we use 4nM of IgG, and 25nM of Fab. In Fig S2E, we use a concentration of Fab fragments double to that of the IgG, to reach an equivalent concentration of binding sites and confirm that the IgG binding we see is indeed due to bivalent binding. In this case, we use 4nM of IgG and 8nM of Fab.

4. Under the section, "Tilting of HA about its membrane anchor contributes to C05 and S139/1 avidity", in the second paragraph, the authors wrote, "If this is correct, we reasoned that avidity could be reduced by constraining tilting of the HA ectodomain. To test this hypothesis, we used FISW84, an antibody that binds to the HA anchor epitope and biases the ectodomain into a tilted conformation (Fig. 4B)."

Can you use some computational models (maybe the same one you used for Figure 4A) to show that when an HA trimer is bounded by FISW84 Fabs, the tilting of HA is constrained? I think this will help substantiate the assertion above.

This is an important point. The model that we employ in Figure 4A is suited to predicting the angles sampled by HAs when they are bound by an IgG antibody, but it does not take into consideration clashes with the viral membrane. It is these clashes that we predict based on published structures (reference 35 in the revised manuscript) will constrain HA tilting when FISW84 binds to the HA anchor. We have revised the text (Lines 247-249) to clarify these points.

5. It would be good if you could mention the strain of HA used in the experiments in Figure 4 in the actual Figure as well (as supposed to just in the figure legend).

We have added this information to Figure 4 in the revised manuscript.

6. I do not see a method section for the structure-based model you used in Figure 4. In the text, you cited your previous study (ref 28) for the model, but it would be good to write about this briefly (and how you specifically apply the model in this study) in this current manuscript.

We have updated the methods to include a subsection (“Geometric Model for Preferred Crosslinking Geometry”) on how the structure-based model was set up, along with a corresponding visual in Fig S3 of the angles of freedom given.

7. In Figure S1 panel D, what is the unit of the antibody concentration? Could you please add it to the graph legend?

We have updated the figure (S1E in the revised manuscript) to include this information.

Reviewer #2 (Significance (Required)):

Previously, this group utilized the same fluorescence-based method to investigate the potency of anti-HA IgG1 antibodies in preventing viral entry versus egress, as well as the tendency of antibodies targeting different HA epitopes to crosslink two HA trimers in cis or in trans (He et al., J Virol, 2024). In this study, they extend their work by evaluating, in-depth, how the density and flexibility of hemagglutinin (HA) on the viral surface influence the binding avidity of anti-HA antibodies. Using two human IgG1 antibodies targeting the HA head, the authors demonstrate that these antibodies can crosslink two HA trimers in cis, even when the trimers are further apart than adjacent HAs. Notably, the study reveals that HA flexibility, rather than density, is the key determinant modulating antibody crosslinking. Even at a 10-fold reduced HA density compared to the original, the antibodies retained their ability to crosslink trimers.

This study provides critical insights into the relationship between HA density, flexibility, and antibody function, adding to the broader understanding of antibody crosslinking-a topic frequently discussed in the field of influenza research. These findings could have significant implications for vaccine design, particularly for strategies involving the display of the HA ectodomain on nanoparticles, potentially guiding the development of more effective influenza vaccines. Furthermore, the broader relevance of these findings may extend to other viruses with similar structural and immunological properties.

My expertise lies in the structural determination of antibody-antigen complexes in influenza and other pathogens. While I may not have sufficient expertise to evaluate specific technical details of the fluorescence-based methods employed, the authors have convincingly demonstrated the robustness of their experimental design and interpretation, supported by appropriate controls.

**Reviewer #3 (Evidence, reproducibility and clarity (Required)):**

SUMMARY
In "Antigen flexibility supports the avidity of hemagglutinin-specific antibodies at low antigen densities", Benegal et al. develop a microscopy-based assay to measure dissociation of HA head-binding antibodies from intact virions. This assay allows the authors to explore the contribution of IgG bivalent avidity to antibody interaction with native virions, which is not accessible using other methods such as BLI. Using this assay, the authors further explore the effect of HA density on IgG avidity with engineered low-HA virions and then with artificial HA-coated microspheres. In addition to measuring antibody dissociation, the authors perform structural analyses to predict the conformational preferences of many HA IgGs from published structures. The authors conclude that low HA densities (down to ~10%) still support high avidity binding for the 2 IgGs tested, and thus there would be little evolutionary pressure for IAV to reduce the HA density as a strategy to evade immune recognition.

MAJOR COMMENTS

The data presented are generally convincing for the two antibodies tested, with some caveats listed below. I believe the microscopy technique is valuable and provides a significant contribution to the field, and I believe that the finding that avidity persists at low densities for IAV is compelling and worth communicating to other virologists. Overall, with the incorporation of the suggested major revisions, this manuscript represents a significant advancement in the field.

A major limitation of the current study is the small number of antibodies tested. Two antibodies are quite few, particularly since this work attempts to generalize these observations with structural predictions of dozens or hundreds of HA antibodies. While I believe that the resilience of IgG binding to lower epitope densities is likely common to many HA antibodies (or antibodies in general), this work alone does not support this. To this end, the authors should acknowledge their limited sample size in the text or discussion and that the generalization to other antibodies is speculative. Alternatively, the authors could demonstrate with additional antibodies (such as F045-092 which is pointed out in Fig S3A and perhaps group 'i' antibodies according to Fig S3A).

This is an important point, and we more explicitly acknowledge this limitation in lines 277-278.

It seems to me lateral diffusion of HA in the viral membrane is an important discussion point that was missed in this manuscript. The authors should comment on what is known about the lateral mobility of HA on virions, and how this could impact the ability of an IgG to crosslink. The authors should comment about whether long range diffusion and/or short range "shuffling" of glycoproteins could contribute to crosslinking preferences of antibodies in addition to the tilt, which is the only movement discussed. As appropriate, the authors should then comment on how this may affect their interpretation of experiments using beads. In experiments on beads, there is certainly no lateral mobility of the HA trimers; what are the consequences of this on the analysis?

We agree that this is an important consideration, and we have revised the manuscript (lines 296-298) to address these points. Briefly, we have previously performed fluorescence recovery after photobleaching of covalently labeled HA and NA on the surface of filamentous influenza particles (10.7554/eLife.43764; see Figure 1B of this reference for a representative example). This data indicates that long range diffusion does not seem to be occurring on the virion surface. Short range diffusion, or shuffling, has not been observed, but cannot be ruled out, and may increase conformations favorable to bivalent binding.

Should the authors qualify the limitations in the scope of their experimental results and the system of choice (beads vs. virions) as described in my previous comments, I suggest three experiments that I believe are essential to support the authors' claims. Alternative to qualifying the limitations, two optional experiments are also listed that could support the authors' claims as they are – those require a more extensive experimental undertaking and are thus labeled [OPTIONAL].

1) The photobleaching experiment shown in Figure S1A. I am concerned that measuring photobleaching in steady state conditions does not properly control for the experimental conditions. In steady state, bleached antibody could unbind and be replaced by fluorescent antibody that has diffused into the field of view. This should be more thoroughly controlled by irreversibly capturing antibody (such as with biotin) and imaging after excess antibody is washed away, or by some other method such as capturing and imaging virus that has been directly labeled with AF555. This should be possible using reagents and techniques already demonstrated by the authors.

We have updated the supplemental information with a more rigorous control for photobleaching; the revised figures are shown in Fig S1A. In this experiment, fluorescent S139/1 IgG was bound to HK68 virions. The antibody was washed away, and the loss of fluorescence signal was imaged separately under two conditions: 1) Dissociation only; an image was collected at 0s and one at 60s. 2) Dissociation and photobleaching; an image was collected at a rate of 1 frame per second for 60 seconds. The difference between the endpoint intensities from both conditions is not statistically significant. This supports our conclusion that, in the absence of antibodies in solution that can exchange with those bound to virions, photobleaching does not make a detectable contribution to the loss of signal we observe in our antibody dissociation experiments.

2) In imaging, the authors analyzed only filamentous virions because they exhibit the best signal to noise ratio, which is a reasonable technical simplification. However, this relies on the assumption that glycoprotein presentation is relatively constant between virions of different sizes. It would be helpful to perform some analysis of small virions in any movie where there is sufficient signal. This would support the assumption that rates for small virions are comparable to those of filaments in the same experiment. This should be possible by performing additional analysis on existing data, without requiring additional experiments.

Thank you for calling our attention to a point that needs clarification. The analysis that was restricted to filaments was for the SEP-HA binding experiments (shown in Fig 3A&B). This was done in order to select only those particles that were not diffraction-limited, so that we could control for any systematic differences in size between the two populations by measuring HA signal per unit particle length. For the dissociation experiments (Fig 2), data was taken from all virions in the fields of view. For this analysis, the normalized dissociation curves were averaged in two ways to account for the potential discrepancy that the reviewer points out. In the first method, the average was taken with each virion equally weighted, while in the second method, the entire field of view was masked and normalized together. Both curves look very similar, suggesting that any potential differences between smaller virions and filaments are not enough to make a quantifiable difference in dissociation rate. A representative dissociation curve with both analyses shown side-by-side has been added in Figure S1B.

3) In figure 3, C05 fab binding is used to assay HA content of the SEP HA virions. An additional method of confirming HA content that is more independent from the imaging assay would be beneficial, such as a Western blot to quantify HA relative to NP, NA, or M1 etc.

We have used western blotting to quantify the amount of HA contained relative to M1 in each population. This new data is discussed in lines 163-168 of the revised manuscript and shown in Figure S2C. As noted in the revised text, western blot analysis suggests that the density of native HA is decreased to ~31% its normal level in SEP-HA virions, lower than the ~75% value determined via fluorescence microscopy. One possible reason for this disparity is the presence of virus-like particles in the SEP-HA sample that completely lack wildtype HA. These would be excluded from our imaging analysis but captured by the western blot.

4) [OPTIONAL] In figure 4, it is depicted that FISW84 biases HA in a tilted conformation, and the authors reasonably propose the reduced flexibility discourages crosslinking by IgGs. From the modeling summarized in Figure S3A, are there any antibodies predicted to prefer crosslinking HA at the same angle FISW84 tilts the ectodomain? Would FISW84 enhance crosslinking by such an antibody?

This is an interesting suggestion, and we have revised the manuscript (lines 247-249) to clarify our thinking on this point. Based on the structure of the FISW84 Fab (PDB ID 6HJQ), we conclude that binding of a single Fab fragment does not necessarily actively tilt the HA ectodomain in a specific direction. Rather, it restricts tilting in the direction that would cause a steric clash between the Fab and the membrane. As a result, HA can still sample a range of angles, but this range is no longer symmetrical about the ectodomain axis. By reducing the likelihood that two HA ectodomains would tilt towards each other at a favorable angle, we would expect all antibodies to be disadvantaged to some degree. A possible exception could be if three FISW84 Fab fragments manage to bind to a single HA trimer. In this case, the HA ectodomain would be forced to remain perpendicular to the membrane to accommodate them all. This would favor antibodies that prefer binding to HAs where the ectodomains are parallel to each other. In our analysis in Figure S3A, this includes primarily antibodies that bind to the HA central stalk, such as 31.b.09. However, we note that these antibodies may encounter barriers to bivalent binding that we do not consider here, including proximity to the FISW84 epitope and the high density of HA in the membrane.

5) [OPTIONAL] In figure S3A, the authors display theoretical tilt and spacing preferences for many HA antibodies based on published structures. Interestingly, their group iii antibody is predicted to prefer greater spacing and tilt, and likewise the authors observe increased binding at lower densities (in figure 3E). It would be beneficial to the work to test group i antibodies (base binding) in the dissociation experiments. The behavior of a base binding antibody, particularly at low densities could reinforce the modeling performed for this work.

This is an excellent suggestion which we are not currently able to pursue for technical reasons. In particular, it would be difficult to distinguish between increased binding of these antibodies at low antigen densities that is due to bivalent attachment (and thus reduced dissociation) versus increased accessibility of the epitope, which may be occluded at higher HA densities.

The experiments are well explained and supported by methods that would enable reproducibility.

The authors state "The statistical tests and the number of replicates used in specific cases are described in the figure legends" yet in many cases this information is absent. For the k values in fig 2D, some indication of error or confidence interval would be helpful.

We have ensured that this information is included in each of the captions. Regarding the k values, formal error propagation is challenging due to the way the k values were derived. Specifically, these values were calculated by fitting the average of the three initial dissociation traces, rather than fitting each replicate individually and then averaging the rate constants. As a result, the usual methods for estimating confidence intervals or standard error of the mean are not directly applicable.

MINOR COMMENTS

o Some of the small details in fig 1A and fig S1 are lost due to small figure size - such as the sialic acid residues and lipid bilayer.

We have resized the figure components.

o Although described in the text, it could be helpful to incorporate into figure 2 why the BLI data is shown for S129 fab. Perhaps indicate in 2C that that curve is "too fast to accurately measure" and perhaps near the table in 2D indicate the blue data is from Lee et al. It may be fine to simply remove the BLI results from the figure and refer to them only in the discussion of the experiments. Even with the measured data, the difference between fab and IgG is striking enough to support the paper, and the BLI data may be more confusing in the figure than it adds.

We have updated the caption for Figure 2D to clarify that binding between the S139/1 Fab and A/WSN/1933 HA is approaching the limit of detection in our assay, and that the additional rates are from Lee *et al.* We have also updated the table to make the presentation of the kinetic parameters more clear.

o In figure 3A, better describe the fluorescent components in the fluorescent images in the legend.

We have updated the caption for Figure 3A to describe the fluorescent components shown in the image. Specifically, the panel labeled ‘HA’ shows signal from a fluorescent FI6v3 scFv, while the panel labeled ‘decoy’ shows signal from the SEP-HA construct.

o From personal experience, the flexibility of HA ectodomain can be significantly affected by how much of the membrane proximal linker region is retained or removed. Could the authors comment on how they chose the cutoff for their HA ectodomain used in the bead experiments and their rationale?

This is an important point, and while the precise impact of the linker on HA flexibility remains uncertain, we agree that it may increase the freedom of motion of the ectodomain relative to the HA membrane anchor. We mention this caveat in the revised text (lines 188-191) and we have added an AlphaFold2 prediction of how our recombinant HA might look to Figure S2D.

o In Figure S1B, if I understand correctly: black dashed line "IgG equivalent dissociation rate" is the experimental data, magenta "Crosslinking model fit" is the theoretically total antibody bound as described by the mathematical model. Then the gray lines "Double- /singly- bound antibodies plot the theoretical amount of antibody bound once and bound twice. If this is correct, I believe it would be clearer if the singly- and doubly- bound were plotted in separate colors, and that this is explained more clearly in the legend.

We have revised the figure to show doubly- and singly-bound curves using different line styles.

o Related to an earlier comment, if lateral diffusion may play a role, how might this differ between different types of antibodies?

As mentioned in our previous response, we do not anticipate that lateral diffusion makes a significant contribution to antibody binding to the surface of virions, although it may be important on the cell surface.

o Could the authors comment in the discussion on how their results on virions may translate to the surface of the infected cell, which is also decorated in viral glycoproteins? Early time points of infection could be an in vivo example of low-density HA. What extent may antibody binding and crosslinking affect viral proteins on the cell surface or the immune response?

This is a very interesting point. Antibody binding to the infected cell surface has been shown to alter viral release and morphology, presumably at lower HA densities than those observed the viral surface. We have added a brief discussion of this point (lines 291-295) to the revised manuscript.

o The github link in the methods is incorrect or not yet available.

Thank you for noting this. We have updated the link.

o Reference 1 has an incorrect or expired link.

These references have been updated.

Reviewer #3 (Significance (Required)):

• This work represents a conceptual advance in our understanding of antibody action on viral pathogens. The authors adapt existing microscopy methodologies to measure antibody avidity in a new way that is better representative of in vivo conditions.

• To my knowledge, this is the first instance of direct measurement of antibody off-rates from intact virus particles, instead of immobilized protein as in BLI, SPR, or interferometry.

• This work should be of interest to virologist and biophysicists interested in the cooperative binding of antibodies and the relation of virus structural organization to antibody recognition. Immunologist may also be influenced by this work. This work may be followed up by other researchers similarly measuring the association and dissociation rates of antibodies with single virions, or otherwise comparing fab to IgG binding to gain insight into when crosslinking is or is not occurring.

• Reviewer expertise: Single-virion imaging, protein complexes, biochemistry, influenza A.

• I do not have sufficient expertise to evaluate the mathematical models and differential equations for modeling the k-on and k-off rates.
